# Need of a formal psychotherapist-delivered counseling as a part of management of bony deformities, with emphasis on clubfoot

**DOI:** 10.4103/0019-5545.74322

**Published:** 2010

**Authors:** Karun Jain, Mruthyunjaya, R. Ravishankar

**Affiliations:** Department of Orthopaedics, JSS Medical College and Hospital, Mysore - 570 004, India. E-mail: leo_karunjain@yahoo.co.in

Sir,

Bony deformities are common but often neglected and under-achieved anonymity in our country. Even though literature endow with standard protocols, the main hurdle that an orthopedician faces during the management is not the deformities per say, it is shattered moral of patients and their relatives which in turn get reflected by patient’s negligent attitude towards the problem like irregular follow-up or even loss of follow-up.

Here we highlight one of the most common encountered bony deformity, Clubfoot or congenital talipes equinovarus (CTEV). Clubfoot is the most common deformity of the bones and joints in newborns. It occurs in about 1 in 1000 babies. Approximately 100,000 infants are born annually with clubfoot, 80% in developing nations.[1–3]

The child with neglected clubfoot is condemned to the downward spiral of deformity, disability, dependency, demoralization, depression, and despair. Digging, plowing, harvesting, and carrying firewood and water are unmanageable tasks for children whose limbs are maimed by heredity, accident, or disease. These children are intellectually capable of integrating into the normal school system but never have the opportunity because their needs are not a high priority. Fewer than 2% of children with disabilities attend school in developing countries. The more difficulty the children experience in locomotion, the less likely they are to attend school. Dr. Ignacio Ponseti, who have done the landmark work for the management of clubfoot, popularly known as Ponseti’s technique, have also highlighted the social stigma and psychological co-morbidity associated with clubfoot.[4,5]

Developing nations such as ours have limited medical and surgical resources. The human cost of neglected clubfoot is enormous, particularly for women and children. Afflicted females are less likely to marry and more likely to suffer abuse. Worldwide, neglected clubfoot is considered to be the most serious cause of physical disability from musculoskeletal birth defects. In agrarian societies, physical disability is a major cause of poverty and ill health. Afflicted individuals are socially and economically disadvantaged, with reduced educational and employment opportunities. The burden of care of the disabled child falls on the mother, who has less time for other children and for domestic, agricultural, and economic activities. Ill health is the most frequent cause and consequence of poverty. The neglected clubfoot deformity results in disability for the individual, a reduced standard of living for the entire family, and a burden to the community. There is an utmost need of various disciplines of medicine to stand together for the management of this crippling problem.[6]

The cause of clubfoot is not exactly known, but it is most likely a genetic disorder and not caused by anything the parents did or did not do.[4,5] Therefore, there is no reason for parents to feel guilty about having a child with clubfoot. It is not a curse on parents or on babies. This is an area which requires to be addressed by the expert of this field. Parents of an otherwise normal infant who is born with clubfoot can be reassured that their baby, when treated by an expert in this field, will have a normal looking foot with essentially normal function. Clubfoot is not always a permanent deformity and the well-treated clubfoot causes no handicap and the individual is fully able to live a normal active life. Parents should be educated specifically that the duration of entire treatment may take few months but the success rate of treatment is well above 90%. Regular and strict time-table follow-up is very much required. Parent’s participation during the course of treatment is as important as the treating doctor.

We emphasize over the need of a regular psychotherapist-delivered counseling sessions as an integral part of regular orthopedic management of clubfoot to improve the current treatment scenario with a better quality of life of clubfoot-affected pediatric population and to educate clubfoot patients about disease, building up their confidence that they can be completely cured and emphasizing on the importance of completing the treatment on time.
